# Perceptions of Interpersonal Versus Intergroup Violence: The Case of Sexual Assault

**DOI:** 10.1371/journal.pone.0112365

**Published:** 2014-11-24

**Authors:** Lisa Droogendyk, Stephen C. Wright

**Affiliations:** Department of Psychology, Simon Fraser University, Burnaby, British Columbia, Canada; Utrecht University, Netherlands

## Abstract

The social identity approach makes a distinction between behavior motivated by intergroup versus interpersonal identities, which may be relevant to victim blaming in the case of rape. Using a mock jury paradigm, we examined the impact of defining rape as an act of interpersonal violence (personal assault) versus intergroup violence (a “hate crime”), crossed with a manipulation describing the attacker as either an acquaintance or stranger. Defining rape in intergroup terms led to less victim blame than when it was defined in interpersonal terms, and participants blamed the victim more when she was assaulted by an acquaintance than a stranger.

## Introduction

A Canadian judge recently made headlines when he showed sympathy to an alleged rapist, pointing out that the victim was wearing revealing clothing and that the rapist could have been “just a clumsy Don Juan” [1]. In another incident, a Toronto police officer suggested women could “avoid dressing like sluts” to reduce their chances of being raped [2]. Incidents like these motivated the women who organized “Slutwalk,” an ongoing international protest against victim blame for rape. It appears that blaming victims of rape remains prevalent, and acceptable to at least some. Considerable social psychological research has investigated the question of why individuals are motivated to engage in victim blaming, with a particular focus on the blame faced by female rape victims. This past research has drawn heavily on perspectives from Belief in a Just World [3] and System Justification Theory [4], which suggest that individuals engage in victim blaming to maintain their view that the world is fair. Victim blaming research has rarely drawn on the social identity approach [5], [6]. Yet, the critical distinction made by the social identity approach between interpersonal and intergroup behaviour may provide a valuable lens for considering people's responses to victims, especially victims of mistreatment or violence by others.

## Interpersonal versus Intergroup Behavior and Victim Blame

The social identity approach [5], [6] suggests that an individual's actions can be guided by either personal or group identities. This basic idea has been shown to be relevant to a wide range of social behaviors and contexts (e.g., [7], [8]), but has typically not been applied to cases of rape or sexual assault (for an exception, see [9]). Although victim blaming research has drawn on ideas from intergroup relations (e.g., stereotyping, sexism), it typically represents rape as a case of *interpersonal violence*, an act perpetrated by one individual upon another individual. Rape is rarely represented as a case of *intergroup violence*, an act perpetrated by a member of one social group upon a member of another group. However, the basic premise of the social identity approach suggests that any act of violence could be interpersonal or intergroup in nature. We extend this analysis by suggesting that assignment of blame for an act of violence may depend upon whether an observer views the violence as interpersonal or intergroup behavior. Specifically, we argue that when rape is viewed as *intergroup violence*, victim blaming may be reduced.

### Social Identity Approach

According to the social identity approach, our behavior is guided by both *personal* and *group* identities. Thus, the participants' behaviour during a given social interaction will fall on a continuum from interpersonal (guided entirely by the actors' personal identities) to intergroup (guided entirely by the actor's group identities). For example, interactions between close friends or romantic partners likely involve primarily *interpersonal* behaviour, meaning that personal identities are the primary determinant of our actions towards them and their actions toward us. Conversely, other interactions are primarily *intergroup*. For example, during team sports the colour of another player's jersey (indicating their group membership) is the primary determinant of our actions towards them and their actions toward us. Military combat provides perhaps the most extreme example, as an individual's personal characteristics become meaningless. Every soldier in the outgroup is simply “the enemy” and violence against them is required.

### Perceptions of Interpersonal Versus Intergroup Violence

This distinction between interpersonal and intergroup behavior may have implications for assigning blame for acts of violence. Rape, in particular, is often represented in North American society as an act of interpersonal violence. For example, like the judge and police officer in the examples above, the news media usually focus on the lives and individual identities of the victim and perpetrator, emphasizing their personal characteristics (e.g., clothing choice, moral characteristics, and behavior). Focusing on these characteristics may lead observers to find the causes of the incident in the individuals involved, thus leading to some degree of victim blame (e.g., [9]–[15]). Conversely, if observers viewed rape from an intergroup perspective, seeing it as perpetrated by a member of one group upon a member of another group, the personal characteristics of the individuals may be less salient and less relevant for judgements of responsibility, leading to reduced victim blame. This view of rape may differ from the “default” representation of rape in contemporary North American society. However, gender categories are certainly part of the prototype of rape and thus people may be open to suggestions that rape could be understood in intergroup rather than interpersonal terms.

### Feminist perspective

This interpersonal versus intergroup representation of rape is also raised by feminist scholars (e.g., [16]–[18]), who have presented rape as a case of men enacting patriarchal control. In fact, several feminist writers have called for rape to be viewed as a hate crime against women, even calling it the “lynching of women” ([19], p. 263). Carney [20] argues that rape meets all the criteria for a hate crime: the victim is selected on the basis of an immutable characteristic (her gender) and the crime incites terror among other members of the target group. Rape, she emphasizes, is “not an act of violence that simply happens to women – it is an act of hate that happens to women because they are women” (p. 320). The implication of the term “hate crime” is that the victim could not have done much to prevent it, given she was selected on the basis of her gender. This view is consistent with our hypotheses based on the social identity approach that representing rape as intergroup violence may reduce the focus on individual identities, making these less relevant for assigning blame. In sum, both the social identity approach and a feminist perspective suggest that the distinction between interpersonal and intergroup violence may have important implications for victim blame.

### Victim-Perpetrator Relationship

One potential moderator of the relationship between the interpersonal versus intergroup representation and victim blaming is the relationship between the victim and the perpetrator. Research suggests that reducing victim blame in the case of an acquaintance rape may be particularly challenging [21], [22]. Although acquaintance rapes are very likely influenced by intergroup factors (e.g., male dominance [18]), it may be more difficult for observers to recognize these when there is an existing relationship between the perpetrator and victim. Viewing rape as interpersonal violence may be the societal and psychological default, and the presence of a personal relationship may serve as a strong cue supporting this default view, decreasing the impact of the intergroup representation. In the case of a stranger rape, these cues to focus on personal identities may be less salient, and thus observers may be more open to viewing the individuals as members of their social groups, strengthening the impact of the intergroup representation. Thus, while representing rape as intergroup rather than interpersonal violence should reduce victim (and perhaps perpetrator) blaming overall, we expect moderation such that this effect might be more pronounced in the case of stranger than acquaintance rape.

### Gender Differences

Past research has demonstrated that for rape cases involving female victims, men report higher levels of victim blame than women ([12], [21], [23], but see also [24], [25]). In addition, consistent with the social identity approach, defining an interaction in intergroup terms may heighten one's identification with the ingroup. This in turn can motivate individuals to protect their ingroup interests by derogating outgroup members. Thus, when rape is defined as intergroup rather than interpersonal, female participants may be particularly likely to show lower victim blame not only because of a reduced focus on the specific individuals but also in order to protect their ingroup. However, men may continue to blame the female victim when the rape is defined in intergroup terms in an effort to support their ingroup.

## Perpetrator Outcomes

Typically, there is an inverse relationship between victim blame and perpetrator blame [12], [21], [22]; as perpetrator blame increases, victim blame decreases. However, this inverse relationship may result from the tendency to see rape in interpersonal terms. That is, observers may be strongly motivated to blame an *individual* and thus to the degree that one individual is seen as more responsible, the other individual is seen as less responsible. If representing rape as intergroup violence leads observers to see the social groups or even the intergroup relationship itself (i.e., the patriarchal nature of society) as a primary cause of rape, then they may assign less blame to both individual victims and individual perpetrators.

## Current Research

A mock jury procedure (e.g., [22], [26]) was used to experimentally examine the impact of representing rape as an act of interpersonal versus intergroup violence for a rape. Participants read one of two “legal definitions” of rape, describing rape as either perpetrated by one individual on another, or by a man against a woman because of her gender. They then read an account of one of two rape cases in which the woman was either acquainted with the rapist, or the rapist was a stranger. Participants then evaluated the blameworthiness of the victim and perpetrator, and provided a decision on whether to convict the alleged perpetrator. Thus, the design included three independent variables: *Legal Definition (Intergroup* versus *Interpersonal*) and *Victim-Perpetrator Relationship (Strangers* versus *Acquaintances)* were manipulated orthogonally, and *Participant Gender* was measured.

### Hypotheses

#### Victim Blame

We predicted a main effect of Legal Definition, such that participants who read a definition of rape as intergroup violence would blame the victim less than those reading a definition of rape as interpersonal violence. However, we also predicted that this effect would be moderated by Victim-Perpetrator relationship, such that the effect of the Legal Definition on victim blame would be stronger when the victim and perpetrator were Strangers compared to when they were Acquaintances. Thus, participants in the Intergroup/Stranger condition should report the least victim blame, while those in the Interpersonal/Acquaintance condition should report the most victim blame, and those in the other two conditions should fall in between these two. Finally, we acknowledged the possibility of a Participant Gender by Legal Definition interaction, such that women's victim blaming may be more impacted by the manipulation of legal definition of rape than men's.

#### Perpetrator Blame

While our predictions for perpetrator blame were tentative, we expected that defining rape as intergroup could lead to lower levels of perpetrator blame compared to defining it as interpersonal.

## Method

### Ethics Statement

The study was approved by the Institutional Review Board at the University of California at Santa Cruz. Participants gave their written consent to participate in the study.

### Participants

Participants were 92 women and 64 men (*M*_age_ = 19.5 years, *SD* = 1.9) recruited from introductory psychology classes in exchange for course credit.

### Procedure

Participants imagined themselves as jurors in a rape trial as they completed two booklets. The first contained instructions, a legal definition of rape and the facts of the case. The second contained scales measuring the dependent variables.

### Manipulations

To focus attention on both manipulations, participants were instructed to closely “consider both the facts of the case and the legal definition of rape when answering any questions or when making decisions.”

#### Legal Definition

Participants first read one of two ostensible legal definition of rape. In the *Interpersonal* condition, rape was defined as “an act of interpersonal violence, an attack on one human being by another human being” and “an assault against a person on the basis of characteristics of that person as an individual.” In the *Intergroup* condition, rape was defined as “an act of intergroup violence: a ‘hate crime’ against women” and “an act of discrimination that is the direct result of prejudices against women as a group.”

#### Victim- Perpetrator Relationship

Participants then read one of two description of the rape case. In both cases, the victim, Marie, was walking to a gas station because of car trouble, when another vehicle stopped and a male driver offered her a ride. In the Acquaintances condition, the description read, “Marie recognized the man driving the car as her friend's brother John.” In the Strangers condition, it read, “Marie did not recognize the man driving the car who introduced himself as John.” After Marie accepted the ride, John turned onto a side road. In all conditions, the facts of the case finished by describing the allegations of the victim, “Marie accuses John of raping her on that side road” and alleged perpetrator's denial, “John maintains his innocence.”

### Measures

*Victim Blame* (α = .77) was measured using an 8-item scale (e.g., “Should Marie be expected to accept any responsibility for what happened?” and “Do you blame Marie at all for what happened?”), with responses provided on 9-point Likert scales. *Perpetrator Blame* (α = .74) was measured using a 6-item scale (e.g., “Should John be expected to accept any responsibility for what happened?” and “Do you blame John at all for what happened?”), with responses provided on 9-point Likert scales.

*Perpetrator Conviction* was measured in a single-item forced choice question which asked participants whether or not they would vote to convict John of rape. [Note: additional open-ended items also asked participants to assign punishments to the perpetrator. However, these items were dropped from the analyses as unclear instructions resulted in unusable data.]

## Results

Data for all dependent variables were analyzed using 2×2×2 ANOVAS, with *Legal Definition (Interpersonal, Intergroup), Victim-Perpetrator Relationship (Strangers, Acquaintances),* and *Participant Gender (Female, Male)* as between-subjects factors.

### Primary Findings

#### Victim blame

Means and standard deviations by condition for Victim Blame can be found in Table 1. All three main effects were significant – Legal Definition, *F*(1, 139) = 8.63, *p* = .004, η^2^ = .05; Victim Behavior, *F*(1, 139) = 10.91, *p* = .001, η^2^ = .06; Participant Gender, *F*(1, 139) = 23.73, *p*<.001, η^2^ = .13.

**Table 1:** Means and standard deviations (in parentheses) by condition and by gender for Victim Blame variable.

			Legal Definition Condition	
			Interpersonal	Intergroup
Victim-Perpetrator Relationship Condition	Acquaint.	Men	4.27 (1.19)	3.44 (1.29)
		Women	2.86 (0.79)	2.44 (1.08)
	Strangers	Men	4.49 (0.98)	3.75 (0.89)
		Women	3.64 (1.34)	3.51 (0.93)

These indicated that participants blamed the victim less in the Intergroup condition (*M* = 3.21, *SD* = 1.16) than in the Interpersonal condition (*M* = 3.72, *SD* = 1.25), blamed the victim more in the Strangers condition (*M* = 3.80, *SD* = 1.11) than in the Acquaintances condition (*M* = 3.14, *SD* = 1.25), and male participants blamed the victim more (*M* = 3.97, *SD* = 1.16) than female participants (*M* = 3.09, *SD* = 1.14). The remaining 2-way and 3-way interactions were non-significant.

### Additional Findings

#### Perpetrator blame

The main effect of Legal Definition was not statistically significant, *F*(1, 142) = 2.99, *p* = .086, η^2^ = .02.

The main effects of Victim-Perpetrator Relationship, *F*(1, 142) = 4.34, *p* = .039, η^2^ = .03, and Participant Gender, *F*(1, 142) = 24.14, *p*<.001, η^2^ = .14 were statistically significant, indicating that the perpetrator was blamed more in the Strangers condition (*M* = 7.72, *SD* = 1.06) than in the Acquaintances condition (*M* = 7.40 , *SD* = 1.13), and female participants (*M* = 7.91, *SD* = 1.16) blamed the perpetrator more than male participants (*M* = 7.06, *SD* = 1.12).

All two-way and 3-way interactions were non-significant.

Overall, Perpetrator Blame and Victim Blame were negatively correlated, *r*(144) = −.49, *p*<.01. Although this correlation was stronger in the Interpersonal condition, *r*(69) = −.51, *p*<.01, than in the Intergroup condition, *r*(73) = −.43, *p*<.01, this difference was not statistically significant, Fischer's *z* = −0.62, *p* = .268 (one-tailed).

#### Perpetrator conviction

The majority of participants (70.3%) indicated a desire to convict the alleged perpetrator. However, gender moderated the likelihood of conviction. Logistic regressions indicated that, compared to men (56.5%), women (80.2%) were significantly more likely to convict the perpetrator (b = 1.16, *p* = .002, odds ratio = 3.190).

There were no significant differences based on Legal Definition (b = −0.56, *p* = .139, odds ratio = 0.57) or Victim-Perpetrator Relationship (b = 0.00, *p* = 1.00, odds ratio = 1.00).

## Discussion

### Review of Findings

#### Victim Blame

The findings provide initial evidence that the degree to which victims of violence will be perceived as responsible for their victimization can be influenced by whether the violence is understood to be intergroup or interpersonal. Specifically, it appears that when rape (traditionally viewed as an act of interpersonal violence in North American society) is viewed as an act of intergroup violence, victim blame may be reduced. Both men and women who read about a rape case preceded by a definition describing rape as intergroup violence blamed the victim less than those who read the same rape case preceded by a definition describing rape as interpersonal violence. The victim blaming literature has focused on situational and attitudinal factors that increase victim blame, but few effective strategies for *reducing* victim blame have been identified. These findings suggest a simple, easily-administered intervention that may reduce victim blame. More broadly, these findings demonstrate at least one way that victims might benefit from a broader adoption of a feminist perspective arguing that rape results from societal norms around the intergroup relations between men and women (e.g., [20], [19]).

Unexpectedly, and apparently inconsistent with previous research (e.g., [21]), we found greater victim blame when participants read that the perpetrator was a stranger, rather than an acquaintance. However, in hindsight, it appears that our specific manipulation of Victim-Perpetrator Relationship may have differed from the vignettes used in most previous research. The majority of these studies use *date rape* scenarios in the “acquaintance” condition, and these studies have consistently shown that women are blamed more in the date rape context, compared to stranger rape. However, the few studies that have examined acquaintance rapes which are *not* date rapes, have yielded inconsistent findings [27]. In these studies, it may be that the other specific circumstances of the case being described may be more important than the victim/perpetrator relationship. The current study also falls in this category. In the current study, in the Strangers condition, Marie accepted a ride from someone she had never met. This may have been seen as a particularly “risky behaviour,” and her apparent “choice” to engage in this behaviour may have led to increased victim blame.

Additionally, the relationship between the victim and perpetrator did not moderate the effect of the intergroup versus interpersonal definition. Although this is inconsistent with our initial hypotheses, this lack of moderation may speak to the utility of the intergroup definition across a variety of contexts and may suggest that it may be valuable to investigate the effectiveness of the intergroup definition in contexts where reducing victim blame is known to be particularly challenging (e.g., a traditional date rape scenario).

Although male participants in our study engaged in more victim blame than female participants, this was not moderated by the interpersonal versus intergroup definition of rape.

Thus, contrary to our tentative social identity-based hypothesis, there was no evidence that men would be particularly likely to derogate the female victim when intergroup concerns were salient. However, it is worth noting that another perspective from the social identity approach, the “Black Sheep Effect” [28], would suggest a competing hypothesis. The Black Sheep Effect suggests that individuals will be particularly likely to derogate ingroup members who flagrantly violate group norms, as an alternative way of maintaining a positive image of the ingroup. According to this perspective, when intergroup concerns are especially salient, male participants may seek to distance themselves from the male perpetrator by showing compassion for the female victim in an effort to restore a view of the ingroup as good. It is possible that both of these opposing processes may have been at play in our study, obscuring a potential interaction between participant gender and the intergroup versus interpersonal definition of rape.

#### Perpetrator Outcomes

Rates of perpetrator blame were consistently high (all means around 7, on a 9-point scale). However, despite these high scores, and consistent with previous research, perpetrators who were strangers were seen as more blameworthy than acquaintances [22]. Opposite to our tentative prediction, participants given an intergroup definition of rape did not blame the perpetrator more than participants given an interpersonal definition. Further, the strong negative correlation between victim blame and perpetrator blame was not significantly attenuated when participants were given an intergroup definition of rape, rather than an interpersonal definition. As it stands, these results suggest that manipulating the representation of rape has a more substantive impact on victim blame than perpetrator blame. Thus, it may be useful for future research to investigate this effect using alternative measures of perpetrator blame. If it is indeed the case that manipulating the interpersonal versus intergroup representation of rape does not affect perpetrator blame, this could be quite positive, as it would mean that victim blame could be reduced without resulting in changes to the perceived responsibility of the (alleged) perpetrator.

### Conclusions

#### Implications for Theory and Future Research

To our knowledge, this is the first study to show that framing an act of violence as interpersonal versus intergroup behaviour can influence victim blaming. This finding represents a novel general extension to the literature on the social identity approach. The social identity approach [5], [6] predicts (and subsequent research has shown) that viewing a situation from an intergroup lens impacts an individual's own motivations, thoughts and actions, and also influences how he/she interprets the actions of other individuals within the intergroup context (e.g., [29], [30]). Recent research suggests that an intergroup frame may also impact the attributions that individuals make for negative events in their own lives - individuals with strong collective identities are less likely to make internal attributions for such events [31]. However, the current research goes a step further by showing that framing events in interpersonal versus intergroup terms also influences how bystanders interpret social interactions that they observe between others. This general finding that the intergroup versus interpersonal representation can be manipulated to influence the evaluation of interactions observed as a bystander may be relevant to other social contexts in which the actors involved could reasonably be viewed as either individuals, or members of two groups. For example, an interpersonal versus intergroup representation might impact how bystanders view interactions involving authority (e.g., police officers interacting with members of the community, or teachers interacting with students), or how they evaluate cross-group helping scenarios.

In this first look at the impact of an intergroup representation on victim blame in the case of rape, we contrasted the intergroup representation with an interpersonal representation, as the interpersonal representation is consistent with the “default” understanding of rape in Western society. However, it is possible that some specific aspect of the interpersonal manipulation, beyond the fact that it represented the crime as an interpersonal act, increased victim blame (i.e., the use of the phrase “characteristics of the individual”). Therefore, we suggest that in future research, investigators could use a more conservative test, in which the intergroup condition is compared with a control condition where participants are not given any definition of rape at all.

#### Applications

This research specifically framed the intergroup definition on rape as “a hate crime against women.” Thus, the results of this study may be relevant not only to rape, but also to legal proceedings surrounding hate crimes more generally. As a case in point, the United States and Canada approach hate crimes differently. In Canada, a person cannot be formally charged with a hate crime. Instead, the accused is charged with a specific crime (e.g., assault, vandalism, murder), and only after he/she is convicted, is it possible for the judge or jurors to consider a hate motivation when deciding on a sentence. The United States allows for a similar option at the time of sentencing, but a person can also be initially charged with a hate crime. The results of the present study provide initial evidence that this difference in procedure may lead to different perceptions of the victim. Formally charging a person with a “hate crime” at the outset of a trial (as in the United States) may lead jurors and other observers (e.g., the media) to view the crime in more intergroup terms, which could reduce perceptions of the victim's blameworthiness. This might lead to different outcomes than a context where the intergroup nature of the crime is not made salient until the trial has concluded (as in Canada).

In sum, the present research has demonstrated that representing rape as an act of intergroup violence, rather than as an act of interpersonal violence, may offer a novel means of reducing victim blame, which may have implications for legal practice and policy.

## Supporting Information

Data S1 **Raw Data File.**
(SAV)
